# SERPINs—From Trap to Treatment

**DOI:** 10.3389/fmed.2019.00025

**Published:** 2019-02-12

**Authors:** Wariya Sanrattana, Coen Maas, Steven de Maat

**Affiliations:** Department of Clinical Chemistry and Haematology, University Medical Center Utrecht, Utrecht University, Utrecht, Netherlands

**Keywords:** SERPIN (serine proteinase inhibitor), protein engineering, bradykinin (BK), hemostasis, therapy

## Abstract

Excessive enzyme activity often has pathological consequences. This for example is the case in thrombosis and hereditary angioedema, where serine proteases of the coagulation system and kallikrein-kinin system are excessively active. Serine proteases are controlled by SERPINs (serine protease inhibitors). We here describe the basic biochemical mechanisms behind SERPIN activity and identify key determinants that influence their function. We explore the clinical phenotypes of several SERPIN deficiencies and review studies where SERPINs are being used beyond replacement therapy. Excitingly, rare human SERPIN mutations have led us and others to believe that it is possible to refine SERPINs toward desired behavior for the treatment of enzyme-driven pathology.

## Introduction

**Figure 1:**
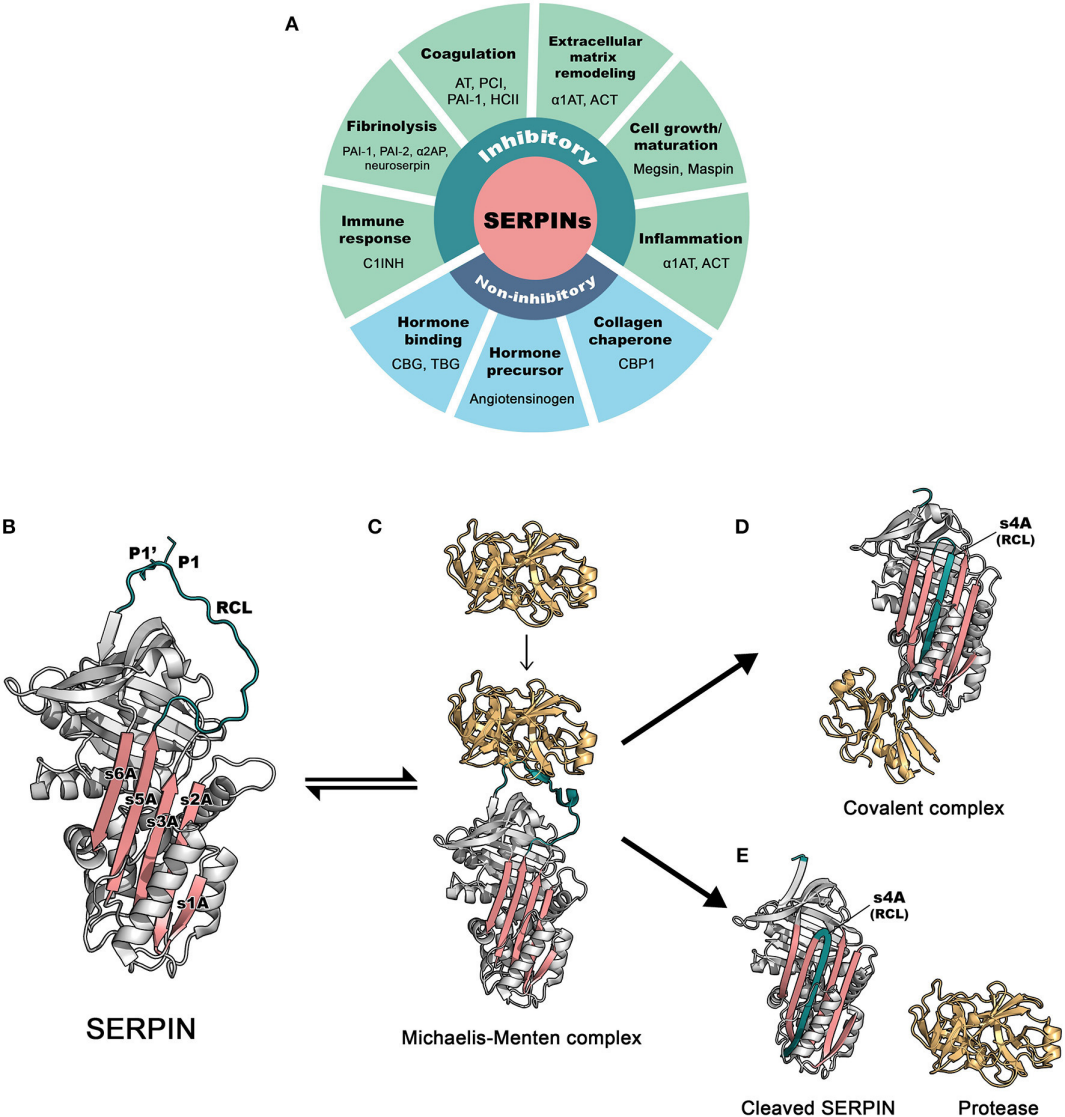
The basic functions and inhibitory mechanism of SERPINs. **(A)** Regulatory functions of SERPINs **(B)** The structure of archetype native α1-antitrypsin. The reactive center loop (RCL) is in green, containing a protease cleavage site (P1-P1′). β-sheet A, comprising of 5 strands (s1A, s2A, s3A, s5A, and s6A) is in pink. These two regions serve as main features, which play an important role in the dramatic conformational change that SERPINs undergo during inhibition. The image was made in PyMol using the PDB file code: 1QLP (3). **(C)** Initially, a target protease docks and binds the recognition site, exposed on the RCL. This step leads to formation of the non-covalent Michaelis-Menten complex [PDB code: 1OPH (4)]. **(D)** Upon cleavage at P1-P1′, the SERPIN spontaneously refolds into a hyperstable conformation, where the N-terminal portion of cleaved RCL is inserted between central β-sheet A. This conformational change of the SERPIN results in “trapping” the covalently linked protease into an inactive form [PDB code: 1EZX (5)]. This SERPIN-protease complex will subsequently be eliminated from the circulation. **(E)** In some cases, a SERPIN can act as a substrate, where protease and SERPIN do not remain covalently linked. This results in an active protease that disassociates from the SERPIN, which leaves the SERPIN in a cleaved form [PDB code: cleaved 7API (6)]. AT: antithrombin; PCI, protein C inhibitor; PAI-1, plasminogen activator inhibitor 1; PAI-2, plasminogen activator inhibitor 2; HCII, heparin cofactor II; α1AT, α1-antitrypsin; ACT, antichymotrypsin; α2AP, α2-antiplasmin; C1INH, C1 esterase inhibitor; CBG, Corticosteroid-binding globulin; TBG, Thyroxine-binding globulin; CBP1, collagen-binding protein 1.

Serine proteases are the “workhorses” of the human body. This enzyme family is conserved throughout evolution. There are 1,121 putative proteases in the human body, and about 180 of these are serine proteases (1, 2). They are involved in diverse physiological processes, ranging from blood coagulation, fibrinolysis, and inflammation to immunity (Figure 1A). The activity of serine proteases is amongst others regulated by a dedicated class of inhibitory proteins called SERPINs (serine protease inhibitors). So far, 37 SERPINs have been identified in the human body. Thirty of these are functional protease inhibitors (7, 8). Human SERPINs are subdivided into 9 subgroups (clade A to I) based on their phylogenetic relationship (9). It is noteworthy that SERPINs are generally capable of inhibiting multiple enzymes. Rather than being considered promiscuous, they appear selective in the sense that the targeted enzymes are often part of a conserved biological mechanism. This for instance is the case for antithrombin (AT), that inhibits multiple enzymes all involved in the coagulation system.

### Structure

SERPINs generally consist of ~ 350–400 amino acid residues, for example, α1-antitrypsin (α1AT) has 394 amino acids. Their molecular weight varies between 40 and 100 kDa due to differences in their glycosylation profile. They are highly expressed in the liver, but are expressed ubiquitously throughout the body (10). SERPINs fold into 7–9 α helices and 3 β-sheets (11). The core structure of SERPINs is highly conserved, which is important for their function. Figure 1B shows the structure of a native SERPIN. Native SERPINs have two main features; (1) five-stranded β-sheet A (s1A, s2A, s3A, s5A, and s6A) are positioned in the middle of the molecule and (2) a flexible reactive center loop (RCL) is positioned on top of the molecule. The RCL contains an enzyme cleavage site (P1-P1′), denoted accordingly to the nomenclature of Schecter and Berger (12), which is located near the C-terminus of the protein sequence.

### Mechanism of Action

SERPINs inhibit target enzymes through a conserved mechanism (13), which involves a unique dramatic conformational change. The nature of native (uncleaved) SERPINs is that they are metastable; i.e., not (yet) in their most stable form. When executing their function, SERPINs act as molecular “mousetraps”, where the RCL is a “bait” and target proteases are “mice” (14). The inhibition process starts when a protease recognizes the bait and binds to the SERPIN by forming a reversible Michaelis-Menten complex (Figure 1C). When the docking protease cleaves the bond between P1 and P1′ residues of the SERPIN, it becomes covalently bound to the main chain carbonyl carbon of the P1 residue of the SERPIN. This cleavage event releases the SERPIN from its metastable conformation (i.e., springing the mousetrap). Hereafter, either the SERPIN remains a stable covalent complex with the enzyme or is used as a “substrate”. In this latter case, the active enzyme dissociates. In 2000, the first SERPIN-protease complex crystallography structure was unveiled (5), confirming the mousetrap-like mechanism of SERPINs. Upon cleavage of the P1-P1′ bond, the C-terminal loop of the SERPIN RCL inserts into the SERPIN body, between its β-sheet A. This leads to the formation of the s4A strand and to a complete antiparallel β-sheet A. When loop insertion is rapid enough, the enzyme active site becomes distorted and inactivated, leaving the enzyme-SERPIN complex covalently bound (Figure 1D) (15, 16). When loop insertion is too slow, the covalent bond is already disrupted before the enzyme active site can be inactivate (4, 17). Now, the SERPIN becomes consumed as a substrate (Figure 1E). The ratio between the two possible pathways is expressed as the stoichiometry of inhibition and should be close to 1 (17) for SERPINs to become powerful inhibitors.

## Key Determinants for SERPIN Functionality

Four features are important for proper SERPIN functionality. Two of these are structural, the other two are sequence-based motifs.

### Reactive Center Loop Mobility

Mobility of the RCL enables loop insertion into β-sheet A after protease cleavage, which is critical for SERPIN stabilization and enzyme inhibition. The N-terminal sequence that precedes the cleavage site (P15-P9), the so-called hinge region, facilitates RCL mobility, and loop insertion (18). Amino acid sequences of alanine-rich hinge region are considerably conserved among inhibitory SERPINs (Table 1).

**Table 1:** Amino acid sequence alignments of human SERPIN reactive center loop.

**SERPIN**	**N-terminal**			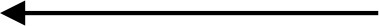								**P4**	**P3**	**P2**	**P1**	**P1′**	**P2'**	**P3'**	**P4'**	** 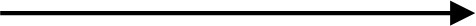 **									**C-terminal**		
**INHIBITORY**																															
SERPINA1	G	T	E	A	A	G	A	M	F	L	E	A	I	P	M	S	I	P	P	E	V	–	–	–	–	K	F	N	K	P	F
SERPINA2	G	T	E	A	T	G	A	P	H	L	E	E	K	A	W	S	K	Y	Q	T	V	–	–	–	–	M	F	N	R	P	F
SERPINA3	G	T	E	A	S	A	A	T	A	V	K	I	T	L	L	S	A	L	V	E	T	R	T	I	V	R	F	N	R	P	F
SERPINA4	G	T	E	A	A	A	A	T	T	F	A	I	K	F	F	S	A	Q	T	T	N	R	H	I	L	R	F	N	R	P	F
SERPINA5	G	T	R	A	A	A	A	T	G	T	I	F	T	F	R	S	A	R	L	N	S	Q	R	L	V	–	F	N	R	P	F
SERPINA9	G	T	E	A	T	A	A	T	T	T	K	F	I	V	R	S	K	D	G	S	Y	F	T	V	S	–	F	N	R	T	F
SERPINA10	G	T	E	A	V	A	G	I	L	S	E	I	T	A	Y	S	M	P	P	V	I	–	–	–	–	K	V	D	R	P	F
SERPINA11	G	T	E	A	G	A	A	S	G	L	L	S	Q	P	P	S	L	N	T	M	S	D	P	H	A	H	F	N	R	P	F
SERPINA12	G	T	E	G	A	A	G	T	G	A	Q	T	L	P	M	E	T	P	L	V	V	K	I	–	–	–	–	D	K	P	Y
SERPINB1	G	T	E	A	A	A	A	T	A	G	I	A	T	F	C	M	L	M	P	E	E	N	–	F	T	A	–	D	H	P	F
SERPINB2	G	T	E	A	A	A	G	T	G	G	V	M	T	G	R	T	G	H	G	G	P	Q	–	F	V	A	–	D	H	P	F
SERPINB3	G	A	E	A	A	A	A	T	A	V	V	G	F	G	S	S	P	T	S	T	N	E	E	F	H	C	–	N	H	P	F
SERPINB4	G	V	E	A	A	A	A	T	A	V	V	V	V	E	L	S	S	P	S	T	N	E	E	F	C	C	–	N	H	P	F
SERPINB6	G	T	E	A	A	A	A	T	A	A	I	M	M	M	R	C	A	R	F	V	P	R	–	F	C	A	–	D	H	P	F
SERPINB7	G	T	E	A	T	A	A	T	G	S	N	I	V	E	K	Q	L	P	Q	S	T	L	–	F	R	A	–	D	H	P	F
SERPINB8	G	T	E	A	A	A	A	T	A	V	V	R	N	S	R	C	S	R	M	E	P	R	–	F	C	A	–	D	H	P	F
SERPINB9	G	T	E	A	A	A	A	S	S	C	F	V	V	A	E	C	C	M	E	S	G	P	R	F	C	A	–	D	H	P	F
SERPINB10	G	T	E	A	A	A	G	S	G	S	E	I	D	I	R	I	R	V	P	S	I	E	–	F	N	A	–	N	H	P	F
SERPINB11	G	T	E	A	A	A	A	T	G	D	S	I	A	V	K	S	L	P	M	R	A	Q	–	F	K	A	–	N	H	P	F
SERPINB12	G	T	Q	A	A	A	A	T	G	A	V	V	S	E	R	S	L	R	S	W	V	E	–	F	N	A	–	N	H	P	F
SERPINB13	G	T	E	A	A	A	A	T	G	I	G	F	T	V	T	S	A	P	G	H	E	N	V	H	C	–	–	N	H	P	F
SERPINC1	G	S	E	A	A	A	S	T	A	V	V	I	A	G	R	S	L	N	P	N	R	V	T	F	K	A	–	N	R	P	F
SERPIND1	G	T	Q	A	T	T	V	T	T	V	G	F	M	P	L	S	T	Q	V	R	–	–	–	F	T	V	–	D	R	P	F
SERPINE1	G	T	V	A	S	S	S	T	A	V	I	V	S	A	R	M	A	P	E	E	I	I	M	–	–	–	–	D	R	P	F
SERPINE2	G	T	K	A	S	A	A	T	T	A	I	L	I	A	R	S	S	P	P	W	–	–	–	F	I	V	–	D	R	P	F
SERPINE3	G	T	K	A	S	G	A	T	A	L	L	L	L	K	R	S	R	I	P	I	–	–	–	F	K	A	–	D	R	P	F
SERPINF2	G	V	E	A	A	A	A	T	S	–	I	A	M	S	R	M	S	L	S	S	–	–	–	F	S	V	–	N	R	P	F
SERPING1	G	V	E	A	A	A	A	S	A	–	I	S	V	A	R	T	L	L	V	–	–	–	–	F	E	V	–	Q	Q	P	F
SERPINI1	G	S	E	A	A	A	V	S	G	M	I	A	I	S	R	M	A	V	L	Y	P	Q	V	I	V	-	–	D	H	P	F
SERPINI2	G	S	E	A	A	T	S	T	G	I	H	I	P	V	I	M	S	L	A	Q	S	Q	-	F	I	A	–	N	H	P	F
**NON-INHIBITORY**																															
SERPINA6	G	V	D	T	A	G	S	T	G	V	T	L	N	L	T	S	K	P	I	I	L	R	N	Q	–	–	–	–	–	P	F
SERPINA7	G	T	E	A	A	A	V	P	E	V	E	L	S	D	Q	P	E	N	T	F	L	H	P	I	I	Q	I	D	R	S	F
SERPINA8	E	R	E	P	T	E	S	T	Q	Q	L	N	K	P	E	V	L	E	V	T	L	N	R	–	–	–	–	–	–	P	F
SERPINB5	G	G	D	S	I	E	V	P	G	A	R	I	L	Q	H	K	D	E	–	–	L	N	A	D	H	–	–	–	–	P	F
SERPINF1	G	A	G	T	T	P	S	P	G	L	Q	P	A	H	L	T	F	P	–	–	L	D	Y	H	L	N	Q	–	–	P	F
SERPINH1	G	N	P	F	D	Q	D	I	Y	G	R	E	E	L	R	S	P	K	–	–	L	F	Y	A	D	H	–	–	–	P	F

Lawrence et al. created a plasminogen activator inhibitor 1 (PAI-1) mutant library, which contains 15 different amino acid substitutions at P14 of PAI-1. Results demonstrate that substitutions with a charged residue at P14, which is normally a small uncharged residue in most of inhibitory SERPINs, significantly retard the inhibitory function of PAI-1 and convert it to a substrate (17). However, the mutations at P14 do not affect protease recognition (19). Therefore, it demonstrates that an uncharged residue is preferable in the hinge region for a proper loop insertion. Remarkably, hinge regions are less conserved among non-inhibitory SERPINs. This suggests that the conserved sequence of the hinge region is important to SERPINs in order to function as inhibitors.

### Reactive Center Loop Length

The length of the N-terminal portion of the RCL is conserved among the members of SERPIN family (Table 1). It has been shown that the length of the RCL critically impacts the kinetic stability of the serpin-protease complex. The length of the RCL, especially the N-terminal portion, should fit the length of β-sheet A to insert in between the sheets during enzyme inhibition. A study by Zhou et al. showed that modifying the RCL length by adding one or two residues dramatically reduced the stability of the complex by up to 1,000,000-fold (20). In contrast, shortening the RCL length by deletion of one or two residues lowered the efficiency of inhibition, but doubled the stability of the complex. Finally, the deletion of more than two residues completely converted the serpin into a substrate.

### Protease Recognition Sequence

In order for a SERPIN to acts as a bait, its RCL contains a sequence motif that is specifically recognized by target enzymes. Interestingly, amino acid sequences adjacent to the cleavage site are highly variable between different SERPINs (Table 1). This variation partially explains their different specificities.

Anderson et al. successfully shifted the target specificity of one of the SERPINs, α1AT through mutagenesis from an inhibitor of neutrophil elastase (an extracellular enzyme) into an inhibitor of furin (an intracellular enzyme). The minimal P4-P1 peptide sequence that is required for recognition and an efficient cleavage by furin is -Arg (R)-X-X-R- (21). Hence, Anderson and co-workers replaced the P4 and P1 residues of the RCL of α1AT, changing it from ^355^AIPM^358^ to ^355^RIPR^358^, and named this variant α1AT-Portland. *In vitro*, the engineered α1AT-Portland exhibited a potent inhibition toward furin and no longer inhibited neutrophil elastase (22).

### Exosites

The specificity of SERPINs is not only determined by their RCL sequences, but also by exosites (23). Exosites are secondary binding sites that are remote from the RCL cleavage site (24). Exosites refine SERPIN specificity in three ways. Firstly, an exosite facilitates a temporary docking site for a target protease, to improve protease binding at P1 residue of SERPIN. For example, when replacing RCL of α1-antichymotrypsin from P6-P3' with that of α1AT, the inhibition rate toward neutrophil elastase, was greatly reduced by 1,500-fold compared to wild-type α1AT (25). This suggests that the SERPIN body selectively contributes to its inhibitory function. Secondly, exosites on extended N- and C-termini assist the binding of target proteases or to specific sites to increase inhibition locally. Alpha 2-antiplasmin (α2AP) uses its C-terminal extension to bind to plasmin, but at the same time uses its N-terminal extension to cross-link to fibrin surface. As a result, α2AP that is cross-linked to fibrin, protects it from degradation. A human single nucleotide polymorphism affects this behavior of α2AP, with functional consequences for the cross-linking of α2AP to fibrin (26). Thirdly, exosites enable interaction with cofactors. The interaction of SERPIN with a cofactor tremendously boosts the inhibition rate of SERPINs and also refines their target specificity. A classic example is the contribution of heparin to the inhibition of coagulation enzymes by antithrombin (AT). The inhibition rate of thrombin by AT is increased by 10,000-fold in the presence of heparin (27). Heparin contains a pentasaccharide sequence that is recognized by the exosites in AT. This induces a conformational change in AT that increases its inhibitory capacity. Furthermore, the bound heparin polysaccharide molecule forms a scaffold that facilitates interaction between thrombin and AT.

## Lessons From Human SERPIN Deficiencies

SERPIN deficiencies show us how SERPINs are involved in physiology.

### Alpha 1-Antitrypsin (α1AT)

Alpha 1-antitrypin is a 52 kDa glycoprotein that strongly inhibits neutrophil elastase. It is encoded by the SERPINA1 gene (28) and is abundantly present in plasma (150–300 mg/dL). Its levels can increase during acute phase reactions. α1AT has a relatively long circulating half-life of 4.5–6 days. By comparison, it is about 3 days, 2.6 days, 1 day, and only 1–2 h for AT, α2AP, C1INH, and plasminogen activator inhibitor-1 (PAI-1), respectively (29, 30). Alpha 1-antitrypin is a powerful protease inhibitor. It inhibits neutrophil elastase at the association rate constant of 6.5 × 10^7^ M^−1^.s^−1^ (31). Functional α1AT deficiency associates with increases risk of pulmonary emphysema and chronic obstructive pulmonary disease (COPD). In this condition, uncontrolled neutrophil elastase activity destructs extracellular matrix components such as collagen and elastin in lung alveolar that leads to remodeling of the lung architecture (32). Replacement therapy is indicated for the treatment of pulmonary disease due to severe α1AT deficiency, along with other pharmacologic therapies such as bronchodilator and anti-inflammatory drugs (33). Alpha 1-antitrypsin is susceptible to pathologic intracellular aggregation as a result of mutations. As α1AT is mainly expressed in the liver, it forms aggregates that accumulate intracellularly in hepatocytes, which consequently leads to liver diseases such as chronic hepatitis, cirrhosis, and hepatocellular carcinoma (34).

### C1 Esterase Inhibitor (C1INH)

C1INH is encoded by the SERPING1 gene. It is a heavily glycosylated glycoprotein (105 kDa; six N- and ten O-glycosylation sites) (35). C1INH inhibits C1s and C1r of the classical complement pathway. It is also a major inhibitor of enzymes in the plasma contact system, i.e., plasma kallikrein (PKa) and activated factor XII (FXIIa) (36).

The clinical phenotype of C1INH deficiency is surprising. Rather than a complement-related disorder, C1INH deficiency causes an overproduction of bradykinin because of an under-regulated contact system. This subsequently leads to hereditary angioedema (37); a disorder characterized by tissue swelling (38). Surprisingly, there is little evidence for excessive intrinsic coagulation, resulting in thrombosis. Plasma-derived C1INH and recombinant C1INH are a treatment of choice for patients with angioedema.

Compared to other SERPINs, C1INH is a relatively poor protease inhibitor, which generally inhibits its targets at the rate constants of about 10^5^ M^−1^.s^−1^. By comparison, other SERPINs such as α1AT, AT, PAI-1, and α2AP have rate constants of about 10^7^ M^−1^.s^−1^ (39). Due to its poor inhibitory capability and short circulation time, high dose infusion is required for C1INH replacement therapy in HAE patients.

Similar to α1AT, some mutations can cause C1INH polymerization and subsequent hepatocellular accumulation (40). In heterozygous patients, the resulting aggregates contain both mutant and wildtype C1INH, as a result of protein-protein interactions. This explains why patients can have plasma C1INH levels below 50%.

### Antithrombin

Antithrombin is a broad inhibitor of blood coagulation proteases. It inhibits thrombin and factor Xa (FXa) and to a lesser extent, factor IXa, XIa, XIIa, PKa, tissue plasminogen activator, urokinase, and plasmin (41–43). AT is encoded by SERPINC1 gene. Low plasma AT levels increases the risk of deep vein thrombosis, pulmonary embolism and ischemic stroke (44). Pharmacological prophylaxis management is only recommended for AT deficient individuals with some clinical circumstances that provoke thrombosis (e.g., surgery, immobility, pregnancy). Current treatment and prophylaxis include low-molecular weight heparin, vitamin K antagonists, plasma-derived or recombinant human AT replacement therapy (45).

## SERPINs as Therapeutic Agents Beyond Replacement Therapy

SERPIN replacement therapies are valuable to restore deficiencies. However, SERPINs have also been studied in animal studies for their therapeutic potential beyond this application. For example, C1INH has been investigated for its therapeutic benefit toward a number of inflammation-related complications. In a porcine model for hemorrhage, a bolus injection of recombinant human C1INH, decreased tissue complement activation and attenuated metabolic acidosis. Furthermore, it reduced circulating tumor necrosis factor α and attenuated renal, intestinal, and lung injury in a dose-dependent manner (46). Pretreatment of Wistar rats with human plasma-derived C1INH exhibited protective effects in ischemia/reperfusion injury of lower extremities and associated lung damage. After 3 h of hind limb ischemia and 24-h reperfusion, C1INH significantly reduced edema formation in the reperfused muscle as well as in the lung, improved muscle viability, and decreased plasma levels of pro-inflammatory cytokines (47).

In clinical studies, administration of C1INH was found to attenuate renal function, but not overall mortality in septic patients (48). In capillary leak syndrome, which may occur secondary to bone marrow transplantation, systemically increased capillary permeability leads to hypertension. Administration of C1INH concentrate improves the overall outcome from 14 (placebo) to 57% over a mean observation period of 9 months after the symptoms (49). Finally, C1INH treatment appeared to confer a benefit in reducing the need for dialysis post-transplant and improved renal function at 12 months post-transplant compared to controls in kidney transplant recipients (50).

### Lessons From α1AT-Pittsburgh

Alpha 1 antitrypsin-Pittsburgh is a rare mutation within the RCL of α1AT. It was first reported in 1,978 and caused severe bleeding episodes in a boy who carried the mutation (51). This single substitution mutation from methionine (M) to arginine (R) at position 358 (M358R), causes a dramatic change in the target specificity of α1AT. α1AT-Pittsburgh is a strong inhibitor of PKa, FXIIa, thrombin, plasmin, and activated protein C (APC), but no longer inhibits neutrophil elastase (52–57).

Alpha 1 antitrypsin-Pittsburgh has been investigated as a therapy for sepsis. In this setting, thrombin and APC, are thought to contribute to cardinal manifestations of gram-negative septicemia, including hypotensive shock and disseminated intravascular coagulation. Recombinant α1AT-Pittsburgh was investigated in a piglet *Pseudomonas aeruginosa* sepsis model (58). Pretreatment with low doses of recombinant α1AT-Pittsburgh attenuates the characteristic decreases in the functional concentrations of AT, FXI, and fibrinogen. In addition, α1AT-Pittsburgh-pretreated group had higher survival rate compared to control. In contrast, in a primate model of *Escherichia coli* sepsis, treatment with recombinant α1AT-Pittsburgh showed no benefit and even exacerbated the associated coagulopathy (59). This unfavorable outcome may have been caused by an overly broad inhibitory spectrum of α1AT-Pittsburgh, which includes inhibition of APC (60, 61). There is little to no experience with the application of α1AT-Pittsburgh in human clinical studies. Most probably, its apparent lack of specificity makes its development as a therapeutic agent unfavorable.

## Designer SERPINs

### Refined Versions of α1AT-Pittsburgh

In order to narrow down the specificity of α1AT-Pittsburgh to FXIIa and PKa, Schapira et al. (53) mutated the RCL of α1AT-Pittsburgh by replacing proline (P) with alanine (A) at the P2 position. As a result, the RCL now imitates the RCL of C1INH, where P2-P1 residues are A and R. This results in a ^357^AR^358^ α1AT mutant. The second-order rate constants show that ^357^AR^358^ α1AT mutant inhibits PKa better than α1AT-Pittsburgh and C1INH by 5.2- and 21.2-fold, respectively. However, this ^357^AR^358^ α1AT mutant inhibits β-FXIIa and thrombin less efficiently (3.8 and 4.9-fold, respectively). This can be explained by the peptide sequence substrate preference of thrombin, which prefers P at P2 position (62). As a consequence, the ^357^AR^358^ α1AT mutant does not prolong the thrombin time in plasma of Wistar rats. Moreover, rats that were pretreated with ^357^AR^358^ α1AT mutant (0.7 mg) were protected from β-FXIIa-induced hypotensive reaction, which is driven by PKa-mediated bradykinin production. A patent application on this invention was filed (US4973668A) in 1990 as a PKa inhibitor. However, to our knowledge, this variant has never been further evaluated for its therapeutic value.

In 2002, Sulikowski et al. (39) developed α1AT-Pittsburgh variants. For this design, they used information from synthetic peptide substrate studies to target PKa and C1s, but not APC. Based on this information, they changed P3-P2 residues of α1AT-Pittsburgh from isoleucine-proline to either leucine-glycine (^356^LGR^358^) or proline-phenylalanine (^356^PFR^358^). The investigators found that the first mutant ^356^LGR^358^ remained a broad-spectrum inhibitor of C1s, PKa, FXIIa, and also APC. However, the second mutant ^356^PFR^358^ showed increased specificity toward PKa, but inhibited all other enzymes less efficiently than mutant ^356^LGR^358^. In other experiments, an additional mutant was developed, based on ^56^LGR^358^ in which the RCL's P4' residue was changed from a P to glutamine (E; ^356^LGRSIPE^362^). However, this additional change did not show beneficial effects.

Another interesting, more recent example of therapeutic SERPIN development is found in the field of hemophilia. In this bleeding disorder, Polderdijk et al. (57) sought to restore the hemostatic balance by developing a strong inhibitor of APC. The investigators chose α1AT-Pittsburgh as a template for the development of a strong APC inhibitor. Hereto, they replaced the P2 and P1′ residues of α1AT-Pittsburgh with a bulky lysine (K) to avoid interaction with thrombin, resulting in a ^357^KRK^359^ α1AT mutant. This mutant specifically inhibits APC, over other coagulation proteases. The ^357^KRK^359^ α1AT mutant has no effects on the PT, aPTT assays or thrombin generation in normal pooled plasma. However, it promotes thrombin generation in plasma from patients with hemophilia A or hemophilia B, indicating its specificity and procoagulant properties. Moreover, the mutant demonstrated efficacy in two different mouse models for hemophilia.

All in all, these studies demonstrate the possible applications of SERPINs beyond replacement therapy. Presently, SERPIN therapies are very costly. For example, the cost of a single-used vial of plasma-derived C1INH is up to $2,300/500 units (63) and the cost of a 2-day treatment with a recombinant AT is approximately $23,000/patient (64). However, these molecules are native protein sequences. The possibility to fine-tune SERPIN specificity and efficacy may reduce the required dosing, thereby potentially lowering the cost. In addition, the use of alternative SERPIN back bones may have dramatic consequences for therapeutic half-life. For example, α1AT has a much longer half-life than C1INH (29, 30).

We expect that an α1AT variant with the inhibitory profile of C1INH will retain this favorable property, enabling cost-effective prophylactic therapy. Finally, liver-specific expression of (designer) SERPINs through gene therapy holds great promise for long-term treatment of enzyme-driven disorders.

## Conclusion

Together, these studies show that engineered SERPINs hold promise for the treatment of a wide variety of diseases. This motivates researchers to find ways to improve this unique class of molecules and extend their application well-beyond disorders in the hemostatic system.

## Author Contributions

WS, CM, and SdM performed literature searches and wrote the manuscript.

### Conflict of Interest Statement

CM is consultant to Shire. CM and SdM are founders of SERPINx BV, a biotech spinout company of University Medical Center Utrecht. The remaining author declares that the research was conducted in the absence of any commercial or financial relationships that could be construed as a potential conflict of interest.
